# Successful Use of OviTex® Bridging Repair in a Super Morbidly Obese Patient With Septic Shock, Perforated Diverticulitis, and Necrotizing Fasciitis of the Abdominal Wall

**DOI:** 10.7759/cureus.97408

**Published:** 2025-11-21

**Authors:** Nicholas J Blount, Eliana Wasserman, Joseph Moorman, Glenn Parker

**Affiliations:** 1 Surgery, Hackensack Meridian School of Medicine, Nutley, USA; 2 Medicine, Jersey Shore University Medical Center, Neptune, USA; 3 Surgery, Jersey Shore University Medical Center, Neptune, USA

**Keywords:** colocutaneous fistula, complex abdominal wall reconstruction, contaminated surgical field, necrotizing fasciitis, super morbid obesity

## Abstract

Managing abdominal wall defects in contaminated surgical fields is particularly challenging in super morbidly obese patients with severe infection. We report the case of a 49-year-old man (BMI 54, 195 kg) who presented in septic shock with necrotizing fasciitis of the abdominal wall secondary to perforated diverticulitis and colocutaneous fistula. Initial management included exploratory laparotomy, extensive debridement of a 25 × 25 cm abdominal wall abscess, anterior resection left in discontinuity, and temporary abdominal closure with an AbThera™ wound vacuum (Solventum, Saint Paul, MN, USA). Once stabilized, the patient underwent definitive repair with the placement of a 25 × 25 cm inlay OviTex® 2S permanent reinforced tissue matrix (manufactured by TELA Bio, Inc., Malvern, PA, USA), the creation of an end colostomy, and wound vacuum placement. Postoperatively, he recovered steadily, was weaned from ventilatory support, tolerated oral intake, and demonstrated functional colostomy output. He was discharged home with a wound vacuum and returned to full function within two months. At six months, he had stable abdominal wall integrity after skin grafting, with plans for staged colostomy reversal and abdominal wall reconstruction after weight loss. This case highlights the successful use of OviTex® in providing both mechanical strength and biologic integration in a high-risk patient where permanent synthetic mesh was contraindicated and biologic mesh alone would have been inadequate.

## Introduction

Managing large abdominal wall defects in a site of active infection is a complex surgical challenge. These scenarios are seen frequently in patients with contaminated fields, such as bowel perforation and necrotizing fasciitis, and are associated with elevated rates of hernia recurrence and wound-related complications [1]. The presence of infection limits the allowance for the use of permanent synthetic mesh. Though synthetic mesh is most durable, it is prone to bacterial colonization and biofilm formation that can lead to further infection and complications [2]. In contrast, bioabsorbable meshes, which are often derived from mammalian tissues, offer a promising alternative due to their reduced infection risk and ability to repopulate and revascularize the host tissue. However, these biological materials are often associated with higher costs and less mechanical strength, making them suboptimal for complicated repairs. OviTex reinforced tissue matrix (RTM) (manufactured by TELA Bio, Inc., Malvern, PA, USA) combines the mechanical durability of a synthetic mesh with an extracellular matrix to facilitate tissue remodeling [3]. It is designed to overcome the limitations of synthetic meshes, particularly the reactions and long-term complications associated with the foreign material, making it an innovative solution for managing abdominal wall defects in infected sites [4].

## Case presentation

**Figure 1:**
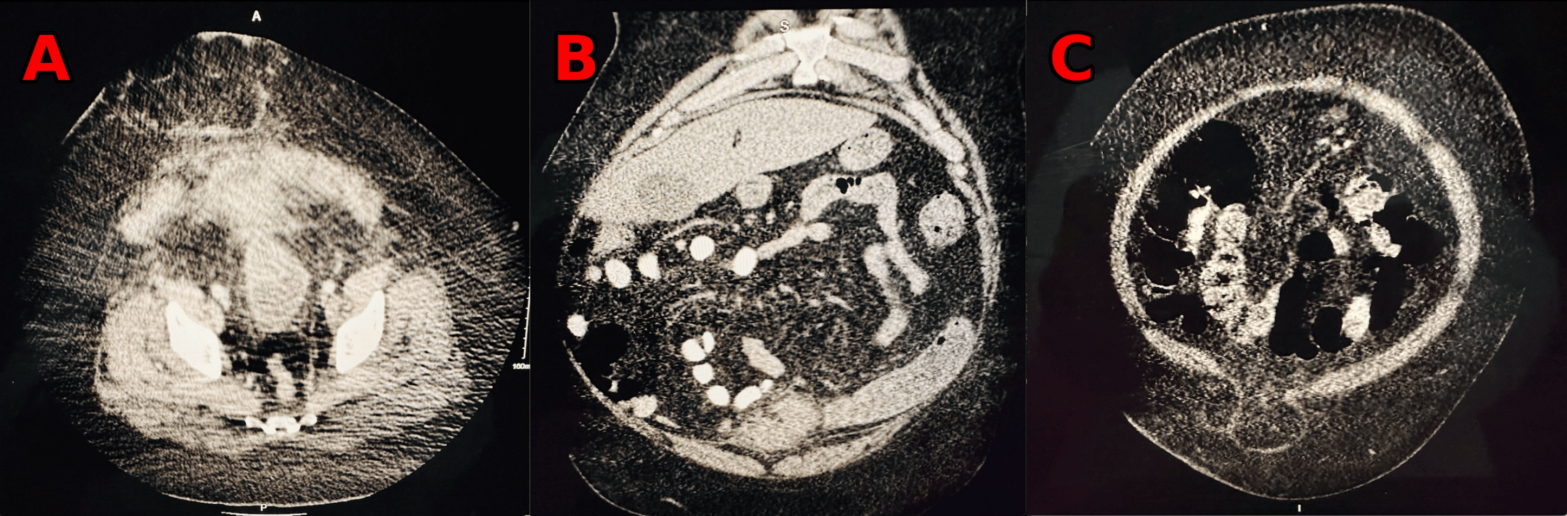
Initial CT scans demonstrating perforated diverticulitis and abdominal wall abscess with necrotizing fasciitis (A) Abdominal CT scan (axial) showing perforated diverticulitis with colocutaneous fistula and necrotizing fasciitis of the abdominal wall. (B) Abdominal CT scan (coronal) showing perforated diverticulitis. (C) Abdominal CT scan (coronal) showing perforated diverticulitis in a ventral hernia

**Figure 2:**
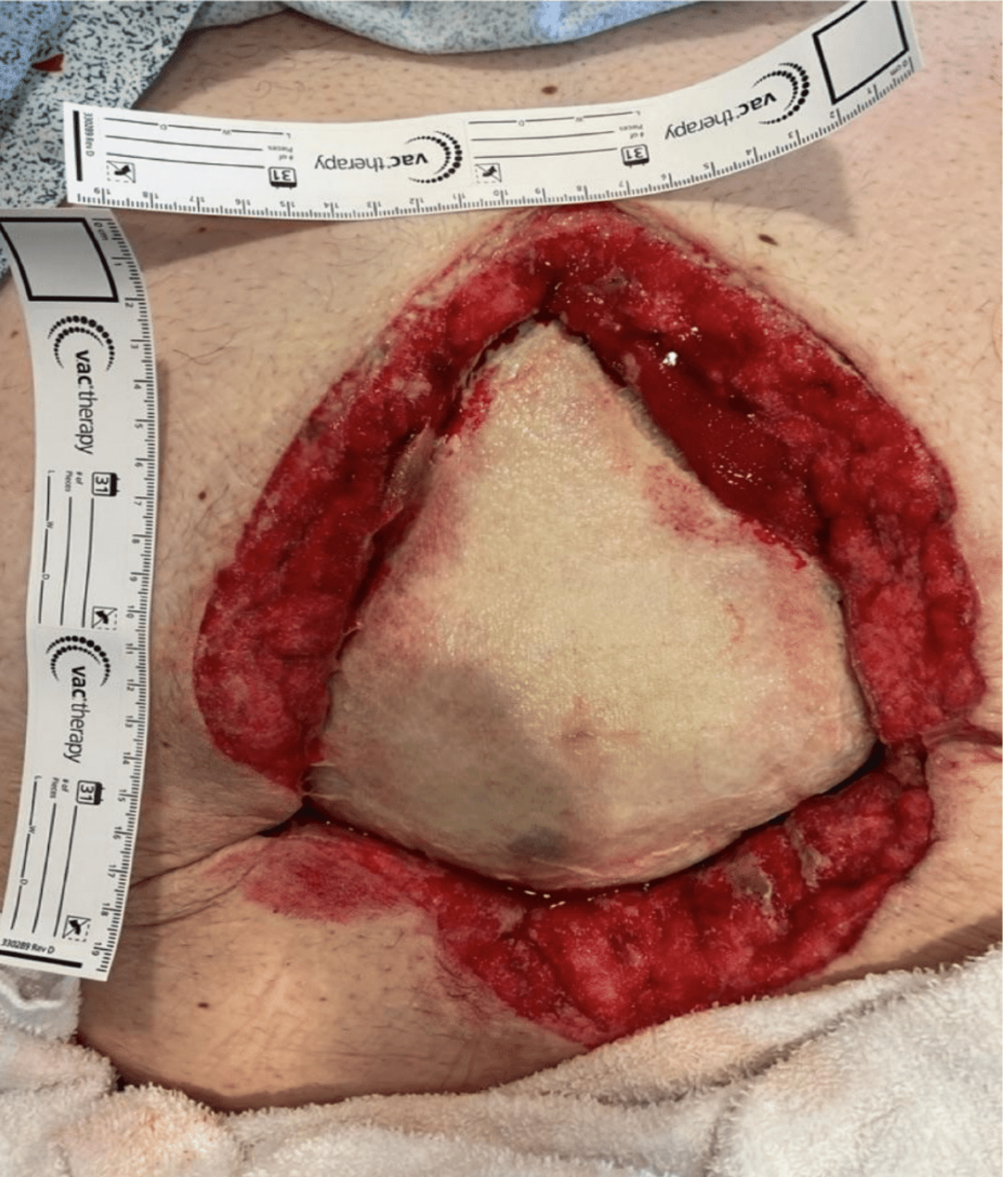
Initial placement of abdominal OviTex®

**Figure 3:**
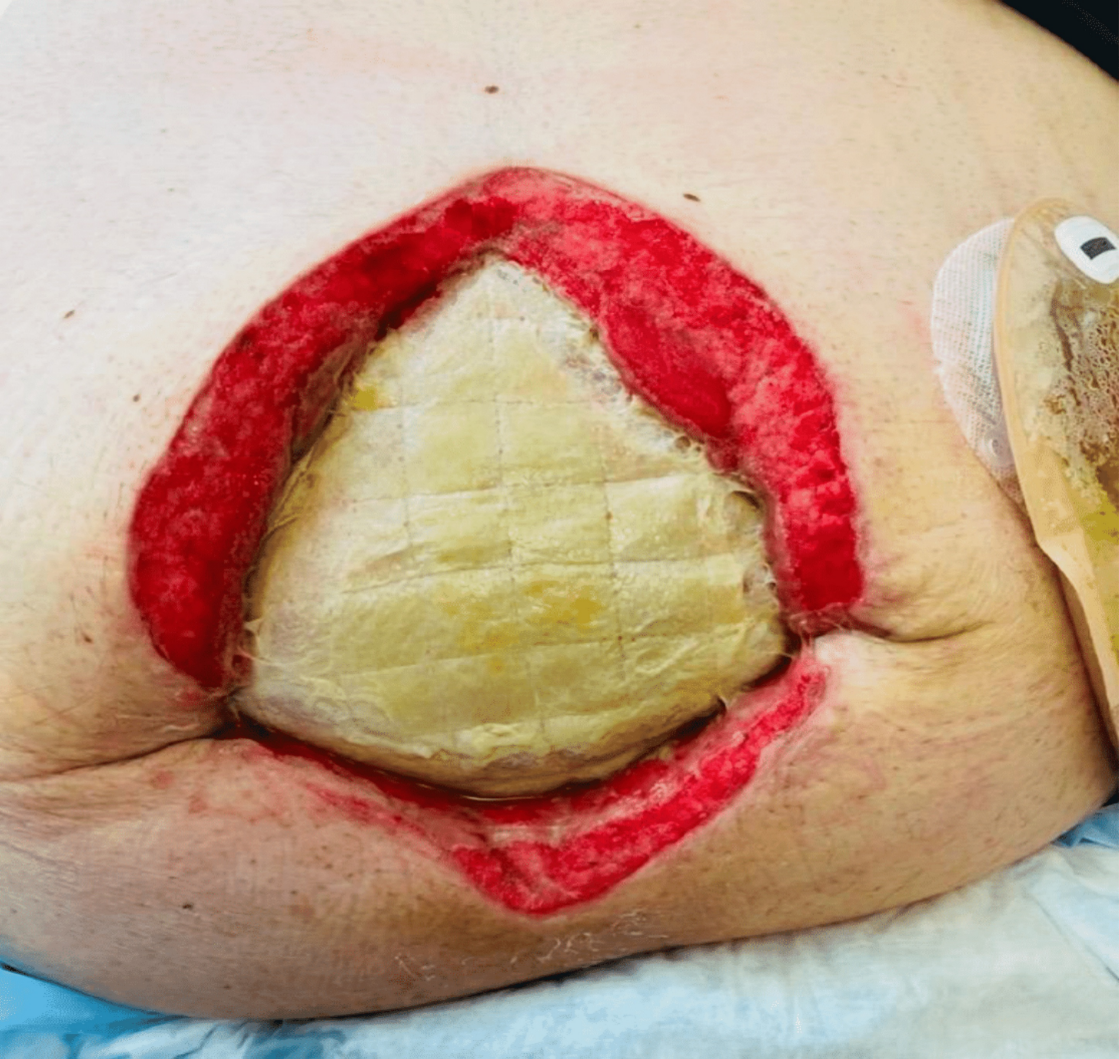
Two months postoperative showing OviTex® RTM intact RTM: reinforced tissue matrix

**Figure 4:**
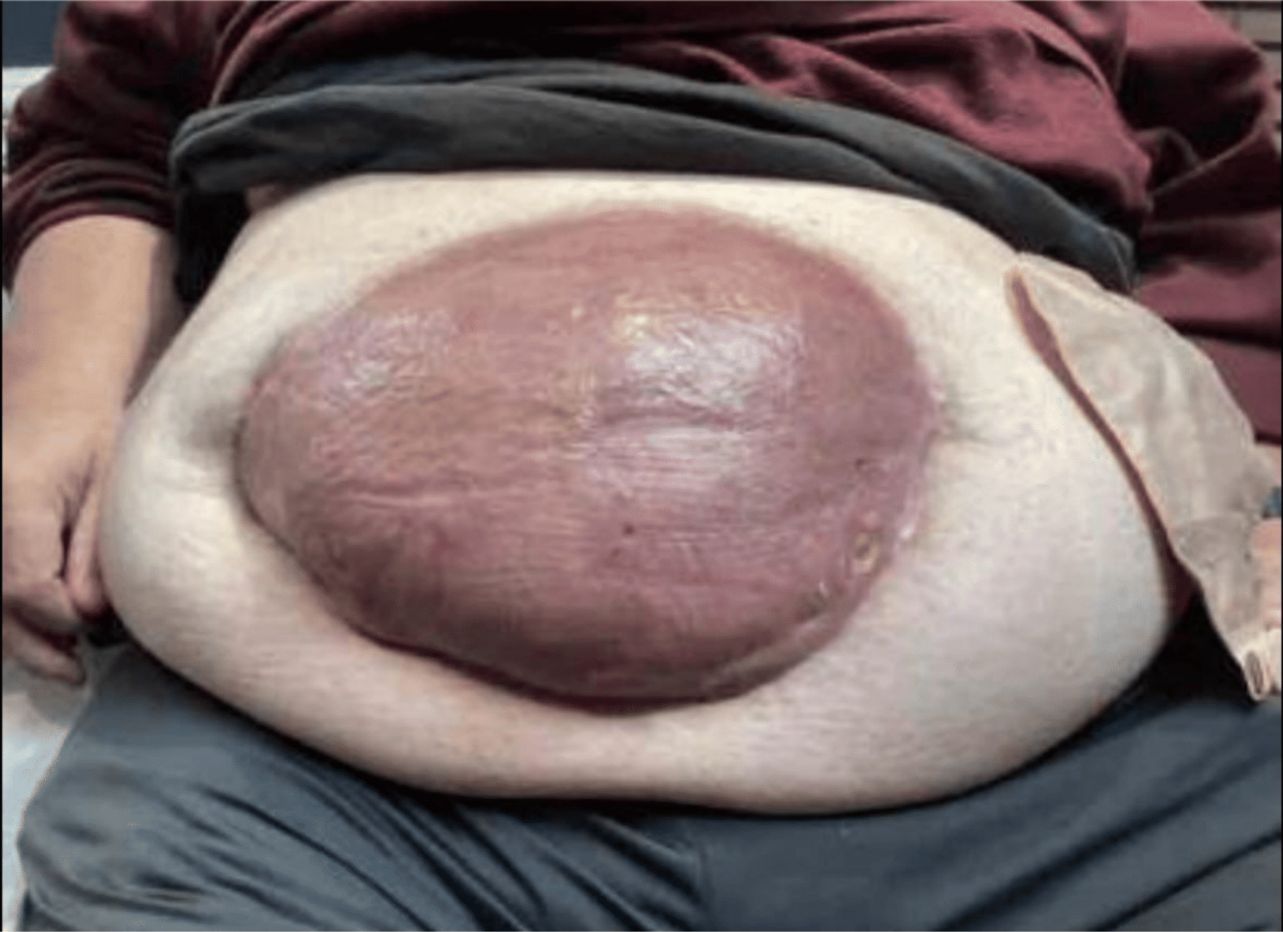
Six months postoperative demonstrating stable abdominal wall integrity after skin grafting

We present the case of a 49-year-old man with super morbid obesity (BMI 54, 195 kg, 430 lbs) who developed septic shock and necrotizing fasciitis of the abdominal wall secondary to perforated diverticulitis with a colocutaneous fistula. The patient initially presented with fever, chills, and abdominal pain. Diagnostic workup confirmed by CT demonstrated perforated diverticulitis with abscess in a ventral abdominal wall hernia (Figures 1-4). The patient was treated with broad-spectrum antibiotics and IV hydration and was placed on nothing by mouth (NPO). Over a 24-hour period, the patient developed a fever of 102°F and tachycardia of 120 beats per minute, with a systolic blood pressure of 90 mmHg and an abdominal examination of guarding, rebound tenderness, and crepitus in the anterior abdominal wall. These progressive clinical findings were highly suspicious for septic shock with necrotizing fasciitis (Laboratory Risk Indicator for Necrotizing Fasciitis (LRINEC) score of 8). His condition had deteriorated requiring emergent operative intervention. The initial procedure was performed as damage control surgery due to hemodynamic instability, the need for pressor support, and fecal peritonitis requiring washout. An exploratory laparotomy was performed with the resection of the sigmoid and rectosigmoid colon, which was left in discontinuity. A 25 × 25 cm anterior abdominal wall abscess with necrotizing fasciitis was also identified, communicating with the sigmoid colon. Cultures were taken along with extensive debridement of the abdominal wall. The abscess cultures ultimately grew *Escherichia coli*, *Klebsiella pneumoniae*, *Streptococcus anginosus,* and *Bacteroides fragilis*. An AbThera™ wound vacuum (Solventum, Saint Paul, MN, USA) was placed with temporary closure and continued resuscitation for damage control surgery. After stabilization for 48 hours, the patient was taken for definitive closure. Due to the anterior abdominal wall abscess resection and retraction of both sides of the incision, a 25 × 25 cm diamond-shaped inlay OviTex 2S permanent RTM bridge was placed, along with the creation of an end colostomy and application of negative-pressure wound therapy for the continued management of the contaminated wound. The patient gradually improved postoperatively, was weaned from ventilatory support, resumed oral intake, demonstrated functional colostomy output, and was ultimately discharged home with a wound vacuum in place. With the inlay RTM in place, the wound was able to granulate and subsequently undergo skin grafting within six months, providing coverage over the hernia defect. There were no complications, intra-abdominal abscess, mesh breakdown, or fistula formation during the phase of healing. The long-term plan is bariatric surgery, gradual weight loss, and reversal of colostomy with abdominal wall reconstruction.

## Discussion

Managing abdominal wall defects in super morbidly obese patients with septic shock, necrotizing fasciitis, and colocutaneous fistula presents significant challenges. This patient has a BMI typically ≥50, which complicates surgical management due to factors such as increased wound complications, poor tissue oxygenation, and difficulties in closing the abdominal wall. Debridement of necrotizing fasciitis often leads to large defects that may be difficult to close primarily. The presence of a colocutaneous fistula further complicates closure due to the increased risk of infection and impaired wound healing. In the case of infection, a temporary abdominal closure, such as the AbThera wound vacuum, may be placed to allow for ongoing resuscitation to ensure continued patient stability before proceeding with the repair. Once the patient is stable, a definitive closure can be performed using mesh [5]. OviTex is an RTM composed of bioabsorbable extracellular matrix material, designed for soft tissue repair such as abdominal wall reconstruction. In this case, OviTex was particularly advantageous due to its bioabsorbability, biocompatibility, and immediate mechanical strength. Unlike permanent synthetic meshes, RTMs are less likely to serve as a nidus for chronic infection, making them ideal for contaminated wounds [4]. The extracellular matrix structure promotes cellular ingrowth, angiogenesis, and tissue repair, supporting the long-term structural integrity of the abdominal wall. OviTex provides immediate mechanical strength to stabilize the abdominal cavity during the healing process [3]. Follow-up considerations for the patient include nutritional support, control of hyperglycemia, control of the ongoing presence of infection, and monitoring for signs of RTM failure or recurrence.

## Conclusions

This case demonstrates that OviTex RTM can provide both immediate strength and biologic integration in the repair of large contaminated abdominal wall defects. In a super morbidly obese patient with septic shock and necrotizing fasciitis, it offered a viable alternative when permanent synthetic mesh was contraindicated and biologic mesh alone would have been insufficient. This case highlights the effectiveness of combining an RTM and NPWT in the management of complex abdominal wall defects, providing a promising approach for restoring abdominal integrity in high-risk patients. Further studies are needed to assess the long-term outcomes and broader applicability of the successful use of OviTex to facilitate closure and recovery.
